# Effect of radiochemotherapy on peripheral immune response in glioblastoma

**DOI:** 10.1007/s00262-024-03722-5

**Published:** 2024-05-16

**Authors:** Léa Hampe, Susy Daumoine, Emeric Limagne, Nicolas Roussot, François Borsotti, Julie Vincent, Sylvia Ilie, Caroline Truntzer, François Ghiringhelli, Marion Thibaudin

**Affiliations:** 1 University Bourgogne Franche-Comté, Dijon, France; 2 Cancer Biology Transfer Platform, Department of Biology and Pathology of Tumors, Georges-François Leclerc Anticancer Center, UNICANCER, 1 rue Professeur Marion, 21000 Dijon, France; 3 Centre de Recherche INSERM LNC-UMR1231, Dijon, France; 4 Department of Medical Oncology, Centre Georges-François Leclerc, Dijon, France; 5 Department of Neurosurgery, University Hospital François Mitterrand, Dijon, France; 6 Genetic and Immunology Medical Institute, Dijon, France

**Keywords:** Glioblastoma, Temozolomide, Steroid, Immune response, Biomarker

## Abstract

**Background:**

Glioblastoma (GBM) is a primary brain tumor with a dismal prognosis, often resistant to immunotherapy and associated with immune suppression. This study aimed to assess the impact of steroids and Stupp-regimen treatment on peripheral blood immune parameters in GBM patients and their association with outcomes.

**Methods:**

Using cytometry panels and bioplex assays, we analyzed the immune phenotype and serum cytokines of 54 GBM patients and 21 healthy volunteers.

**Results:**

GBM patients exhibited decreased lymphoid cell numbers (CD4, CD8 T cells, NKT cells) with heightened immune checkpoint expression and increased myeloid cell numbers (especially neutrophils), along with elevated pro-inflammatory cytokine levels. Steroid use decreased T and NK cell numbers, while radio-chemotherapy led to decreased lymphoid cell numbers, increased myeloid cell numbers, and heightened immune checkpoint expression. Certain immune cell subsets were identified as potential outcome predictors.

**Conclusion:**

Overall, these findings shed light on the peripheral immune landscape in GBM, emphasizing the immunosuppressive effects of treatment. Baseline immune parameters may serve as prognostic indicators for treatment response.

**Supplementary Information:**

The online version contains supplementary material available at 10.1007/s00262-024-03722-5.

## Background

Glioblastoma (GBM) is the most frequent and most aggressive primary brain tumor. Treatment is based on surgery, consisting of complete removal of the gross tumor mass whenever possible. Following surgery, treatment is pursued with a combination of radiotherapy plus chemotherapy (temozolomide), followed by six months of adjuvant temozolomide. Despite this aggressive therapy, almost all patients present recurrence. However, few therapies are currently available to treat progressive disease, and available options offer no benefit in terms of overall survival, with significant toxicities [1, 2]. Actually, only tumor-treating fields, an antimitotic treatment modality that interferes with glioblastoma cell division and organelle assembly by delivering low-intensity alternating electric fields to the tumor, have recently demonstrated a modest ability to improve patient survival [3]. Classical prognostic factors include complete tumor resection, methylation of the MGMT (O6-MethylGuanine-DNA MethylTransferase) promoter, and the use of steroids [4–9].

GBM is associated with crossing of the blood–brain barrier and is widely invaded by bone marrow-derived myeloid cells. In addition, some T cells are also found in the core of the tumor and high infiltration by T cells is associated with better outcome, suggesting that immunotherapy could be a tool to improve treatment of this devastating disease [10, 11]. Several ongoing clinical trials are testing this hypothesis [12–14]. These include therapeutic vaccines [15], T cell therapy with chimeric antigen receptors [16] and immune checkpoint inhibitors [17], with an active search for new pathways as potential targets for immunotherapy [18]. However, first results from clinical trials of immune checkpoint inhibitors have shown disappointing results, with no improvement in overall survival in a phase III second-line trial following failure of radio-chemotherapy [19]. Several hypotheses may explain these results. The leading hypothesis is the immunosuppressive context of GBM. Firstly, GBM can induce sequestration of immune cells in the bone marrow, leading to lymphopenia and a reduced ability to generate an immune response [20]. Secondly, temozolomide is a strong lymphopenic agent and can induce a substantial decrease in the number of CD4 T cells in the blood, thereby leading to a state of immunosuppression [21, 22]. Thirdly, the use of steroids induces lymphopenia by directly killing activated T cells, but also by impeding their capacity to produce cytokines and thus induce an effector immune response [23, 24].

In contrast, it has been shown that chemotherapy-induced myeloid and lymphoid depletion induces IL-2, IL-7, and IL-15 production, and mediates homeostatic proliferation and immune reconstitution, thereby promoting an immune response [25]. Studies in mice [26, 27] and humans [28, 29] have shown that the temozolomide-based myeloablative regimen enables large-scale vaccine-specific immune responses and persistence of chimeric antigen receptor T cells [30]. Radiation could also promote immunogenic cell death. This phenomenon triggers optimal antigenic presentation and improves the T cell-dependent anti-tumor immune response [31].

In this study, in order to determine the impact of steroids and radio-chemotherapy on the systemic immune response of patients with GBM, we prospectively analyzed the number, phenotype, and functionality of different immune cell populations during radio-chemotherapy and correlated this information with clinical prognostic factors; such as steroid use and MGMT methylation status, as well as with response to treatment.

## Methods

### Study participants

This study included stage IV glioblastoma patients treated by surgery and a Stupp protocol at the Georges Francois Leclerc Center, Dijon, France, between May 2018 and April 2020. Written informed consent was obtained from all patients before enrolment. The hospital institutional review board approved the study in accordance with the principles of Good Clinical Practice, the Declaration of Helsinki, and other applicable local regulations. This study falls within the scope of the biobanking authorisation registered under the registration number AC-2019-3531. As a control group, we used healthy blood donors from the Etablissement Français du Sang, matched on age and sex to GBM patients.

Patients received concurrent chemoradiotherapy and adjuvant chemotherapy according to the original protocol proposed by Stupp et al. [32]. Temozolomide (75 mg/m^2^) was administered on days 1 through 42 with concomitant radiotherapy (60 Gy). After four weeks, treatment was pursued by the administration of temozolomide alone (150–200 mg/m^2^) on days 1–5 in six consecutive 4-week cycles or to progression. Response to treatment was evaluated based on regular follow up with MRI scanning. The first post chemo-radiotherapy MRI was usually ordered 4–6 weeks after the last radiotherapy session, followed by regular MRI every three months unless clinically indicated for earlier examination. MRI were visually evaluated by the radiologist. Unclear findings were reviewed by a multidisciplinary neuro-oncology tumor board, mostly with a recommendation for an earlier control exam.

O6-Methylguanine-DNA methyltransferase (MGMT) promoter methylation status was determined after DNA extraction, bisulfite conversion and RT-qPCR [33].

For each patient, five blood draws were taken at three different timepoints, namely: before the start of treatment (C1), after 14 days of treatment (C2) and after 28 days of treatment (C3). Plasma and peripheral blood mononuclear cells (PBMC) from these samples were collected and stored, and the analyses of myeloid and lymphoid populations and of lymphocyte function were performed by flow cytometry.

### Study of the specific T response of TERT by the ELISpot technique

#### Specific stimulation by TERT-derived peptides

For PBMC-specific stimulation, a pool of four UCP peptides (Universal Cancer Peptides) derived from human telomerase was used: UCP1 (PAAFRALVAQCLVCV), UCP2 (KSVWSKLQSIGIRQH), UCP3 (GTAFVQMPAHGLFPW), and UCP4 (SLCYSILKAKNAGMS). They have previously been described [34] and were purchased from ProImmune (at 90% purity). Before use, the peptides were reconstituted in 1X PBS containing 10% DMSO to a concentration of 4 mg/mL, before being aliquoted and frozen at is −80 °C.

#### Pre-stimulation of PBMC with selected UCP peptides

PBMC suspensions for each patient were thawed and diluted in RPMI 1640 containing 5% of decomplemented FBS. After washing the cells, the isolated PBMC were counted with trypan blue and resuspended in the RPMI 1640 5% decomplemented FSB medium at a rate of 4.10^5^ cells per well in a 24-well culture plate. For each patient, and their samples (C1, C2, and C3), two conditions were performed: a negative control, without any peptide, and a test well in which the pool of the four UCP peptides was added at a final concentration of 5 μg/mL. After 24 h of culture we added recombinant human IL-7 (130-093-93, Miltenyi Biotec) to all wells at a final concentration of 5 ng/mL, and after three days of culture, we added recombinant human IL-2 (130-097-743, Miltenyi Biotec) at a final concentration of 20 IU/mL [34, 35].

#### Measurement of TERT specific T response by ELISpot technique

For this study, we aimed to measure TERT tumor antigen-specific T-cell immunity using an IFN-γ ELISpot assay (hIFNgp-2 M/5, Immunospot). To perform the ELISpot assay, we first counted live leucocytes in the cultured cells by flow cytometry (CytoFLEX Cytometer, Beckman Coulter): a small volume of cell suspension from each condition was labelled with an anti-CD45 antibody APC-Violet750 (A79392, Beckman Coulter) and DAPI (130-111-570, Miltenyi Biotec). Unlabelled cells were then plated on a membrane coated with an IFN-γ-specific capture antibody at a rate of 1.10^5^ cells per well in a final volume of 200 μL of CTL medium (hIFNgp-2 M/5, ImmunoSpot) + 1% L-glutamine (G7513, Sigma). Three conditions were performed for each sample: with cells derived from the wells that were not supplemented with UCP peptides, the negative and positive controls. These cells were cultured in the ELISpot plate with CTL medium + 1% L-glutamine for the negative control and for the positive control, 1 μg/mL ionomycin (19,657, Sigma) and 50 ng/mL Phorbol Myristate Acetate (PMA, P1585, Sigma) were added to the wells. Cells that were stimulated by UCP peptides upon activation were cultured in the ELISPot plate with 5 μg/mL of UCP peptides. The cells were then incubated for 24 h at 37 °C, and the spots were revealed following the manufacturer’s instructions. The ELISpot assay was read on an ELISpot plate reader (Immunospot S6 Alpha) allowing detection, analysis and counting of spots.

### Cytometry analysis

#### Lymphoid and myeloid population identification

Staining protocol: 100 μL of total heparinized blood was added to each DURAClone tube containing liquid antibodies to lymphoid and myeloid panels, vortexed immediately for 15 s and incubated for 10 min at room temperature in the dark. Two millilitres of red blood lysis solution (VersaLyse solution, A09777, Beckman Coulter) containing 50 μL of the fixative agent IOTest 3 Fixative solution (A07800, Beckman Coulter) was added, inverted and incubated for 15 min in the dark. After centrifugation and washing with 3 mL of PBS 1X, cells were resuspended in 150 µL PBS 1X before acquisition on a CytoFLEX cytometer (Beckman Coulter). The gating strategies are described in Supplementary Figs. 6 and 7.

#### Lymphocyte function analysis

Staining procedure: 50 μL of total heparinized blood was transferred into a DURactive 1 tube (C11101, Beckman Coulter) for 3 h at 37 °C in the dark. After activation, 25 μL of PerFix-NC R1 buffer (PerFix-NC kit, B31168, Beckman Coulter) was added on vortex and incubated for 15 min at room temperature. Then, 2 mL of PBS 1X was added, and after centrifugation, the pellet was resuspended in 25 μL of FBS (Dutscher) and 300 μL of PerFix-NC R2 buffer was added. A 325 μL aliquot was transferred to a DURAClone tube containing the liquid antibody, vortexed immediately for 15 s and incubated for 1 h at room temperature in the dark. PBS 1X (3 mL) was added to the tubes, incubated for 5 min at room temperature in the dark before centrifugation for 5 min at 500 g. After supernatant removal, the cells were resuspended in 3 mL of 1X PerFix-NC R3 buffer before another 5 min centrifugation at 500 g. The pellet was dried and resuspended in 150 μL of 1X R3 buffer. Acquisition was done on a CytoFLEX cytometer. The gating strategy is described in Supplementary Fig. 8.

All cytometry analyses were done with Kaluza 1.3 software (Beckman Coulter).

### Statistical analysis

Progression-free survival (PFS) was defined as the time from the date of surgery to the first recorded evidence of disease progression according to RECIST criteria, clinical assessment or death. Overall survival (OS) was calculated as the time from the date of surgery to the date of death. The median follow-up was calculated using the reverse Kaplan–Meier method, and survival endpoints are described using the Kaplan–Meier method. Data for patients who were alive and event-free were censored after one year of follow-up after the start of treatment. Survival probabilities were estimated using the Kaplan–Meier method, and survival curves were compared using the log-rank test.

Statistical analyses were performed using Prism GraphPad software [not significant (ns), *, *p* < 0.05; **, *p* < 0.01; ***, *p* < 0.001; and ****, *p* < 0.0001]. Results are shown as the mean ± SD. Datasets were compared using an unpaired Mann–Whitney–Wilcoxon test. No statistical corrections were performed.

## Results

### Influence of glioblastoma on peripheral blood immune phenotype and serum cytokines

Patient and control characteristics are detailed in Tables 1 and 2. We included 54 patients, and 21 sex-and age-matched, untreated, healthy volunteers as controls. Thirteen patients underwent diagnostic biopsy, 15 patients underwent macroscopic complete tumor resection and 26 patients underwent partial tumor resection. Seventeen patients had a methylated MGMT promoter and five patients had IDH1/2 mutation. All patients had grade IV glioma according to the 2021 WHO brain tumor classification.

**Table 1:** Patients’ characteristics

Characteristics	(N = 54 patients)
*Sex*	
Male-no. (%)	33 (61.1)
Female-no. (%)	21 (38.9)
*Age*	
N	54
Mean (std)	60.6 (12.6)
Median [min–max]	62.0 [31.0–82.0]
*Tumor localisation*	
Frontal-no (%)	18 (33.3)
Temporal-no. (%)	7 (13.0)
Temporo-parietal-no. (%)	4 (7.4)
Parietal-no (%)	7 (13.0)
Occipital-no (%)	3 (5.6)
Other-no (%)	15 (27.7)
*MGMT*	
Methylated-no (%)	17 (31.5)
Unmethylated-no. (%)	26 (48.1)
NC-no (%)	11 (20.4)
*IDH 1,2*	
Mutation-no. (%)	5 (9.3)
Wild type-no. (%)	48 (88.8)
NC-no (%)	1 (1.9)
*Extent of resection*	
Biopsy-no (%)	13 (24.1)
R0-no. (%)	5 (9.3)
R1-no. (%)	10 (18.5)
R2-no (%)	26 (48.1)
*Corticosteroids at baseline*	
Yes-no. (%)	30 (55.6)
No-no. (%)	24 (44.4)

**Table 2:** Healthy volunteers’ characteristics

Characteristics	(N = 21 patients)
*Sex*	
Male-no. (%)	12 (57.2)
Female-no. (%)	9 (42.8)
*Age*	
N	21
Mean (std)	53.7 (7.1)
Median [min–max]	51.0 [42.0–69.0]

**Fig. 1:**
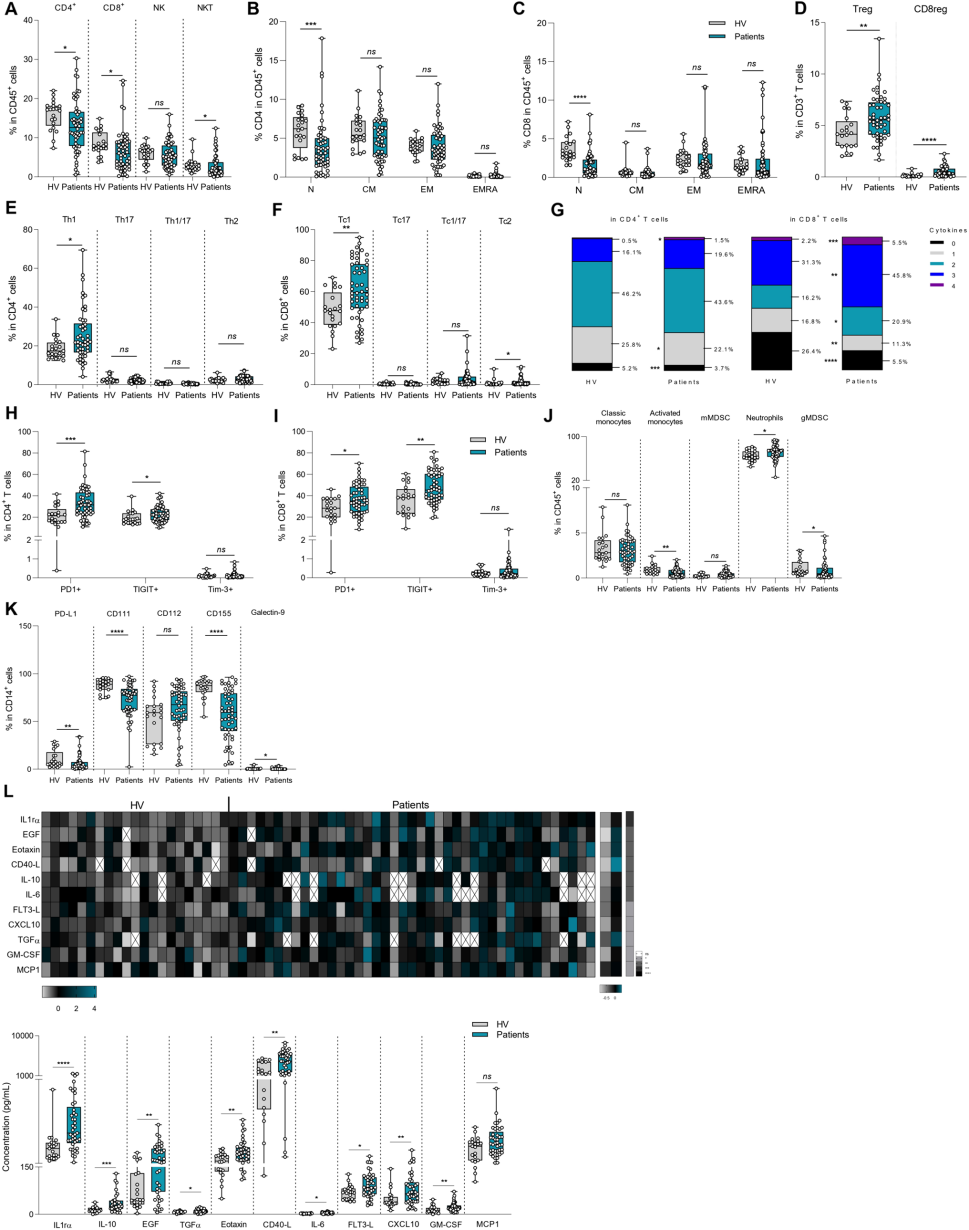
Influence of glioblastoma on peripheral blood immune phenotyping and serum cytokines. Blood samples from healthy volunteers (n = 21) and baseline glioblastoma patients (n = 54) were activated or not, stained and then analyzed by flow cytometry (**A**–**K**). **A** Box plot showing the percentage of different lymphoid populations in CD45^+^ cells: CD4^+^ T cells (CD45^+^ CD3^+^ CD4^+^), CD8^+^ T cells (CD45^+^ CD3^+^ CD8^+^), Natural Killer (NK) cells (CD45^+^ CD3^−^ CD56^+^) and Natural Killer T (NKT) cells (CD45^+^ CD3^+^ CD56^+^). **B** Box plot showing the proportion of different stages of CD4 T cell differentiation in CD45^+^ cells: naïve cells « N» (CD4^+^ CD45RA^+^ CCR7^+^), effector memory cells « EM» (CD4^+^ CD45RA^−^ CCR7^−^), central memory cells « CM» (CD4^+^ CD45RA^−^ CCR7^+^) and effector memory cells re-expressing CD45RA « EMRA» (CD4^+^ CD45RA^+^ CCR7^−^). **C** Box plot showing the proportion of different stages of CD8 T cell differentiation in CD45^+^ cells: naïve cells « N» (CD4^+^ CD45RA^+^ CCR7^+^), effector memory cells « EM» (CD4^+^ CD45RA^−^ CCR7^−^), central memory cells « CM» (CD4^+^ CD45RA^−^ CCR7^+^) and effector memory cells re-expressing CD45RA « EMRA» (CD4^+^ CD45RA^+^ CCR7^−^). **D** Box plot showing the percentage of regulatory CD4 T cells (CD4^+^ Foxp3^+^) and regulatory CD8 T cells (CD8^+^ Foxp3^+^) in T lymphocytes CD3^+^. **E** Box plot showing the proportion of different CD4 T cell subpopulations in CD4^+^ cells: Th1 (CD4^+^ FoxP3^−^ IFNγ^+^ IL-17A^−^), Th17 (CD4^+^ FoxP3^−^ IFNγ^−^ IL-17A^+^), Th1/17 (CD4^+^ FoxP3^−^ IFNγ^+^ IL-17A^+^) and Th2 (CD4^+^ FoxP3^−^ IFNγ^−^ IL-17A^−^ IL-4^+^) cells. **F** Box plot showing the proportion of different CD8 T cell subpopulations in CD8^+^ cells: Tc1 (CD8^+^ FoxP3^−^ IFNγ^+^ IL-17A^−^), Tc17 (CD8^+^ FoxP3^−^ IFNγ^−^ IL-17A^+^), Tc1/17 (CD8^+^ FoxP3^−^IFNγ^+^ IL-17A^+^) and Tc2 (CD8^+^ FoxP3^−^ IFNγ^−^ IL-17A^−^ IL-4^+^) cells. **G** Representation of the polyfunctionality of CD4^+^ (left) and CD8^+^ (right) T cells through analysis of their ability to secrete 0, 1, 2, 3, or 4 different cytokines (IFNγ, TNFα, Granzyme B and IL-2). **H, I** Box plot showing the percentage of CD4^+^ (h) or CD8^+^ (i) T cells expressing 3 immune checkpoints PD-1, TIGIT or Tim-3. **J** Box plot showing the frequency of myeloid subpopulations in CD45^+^ cells: classical monocytes (CD11b^+^ CD15^−^ CD14^+^ HLA-DR^+^), activated monocytes (CD11b^+^ CD15^−^ CD14low HLA-DR^+^), monocytic MDSC (CD11b^+^ CD15^−^ CD14^+^ HLA-DR^low^), neutrophils (CD11b^+^ CD15^+^ CD14^−^) and granulocytic MDSC (CD11b^+^ CD15^+^ CD14^+^). **K** Box plot showing the frequency of PD-L1^+^, CD111^+^, CD112^+^, CD155^+^ and Galectin-9^+^ populations within monocytes (CD14^+^). **L** Cytokine secretion in the plasma of healthy volunteers and patients was analyzed using a bioplex technique. Top: the heatmap on the left corresponds to normalized cytokine expression (z-score) for which there is a statistically significant difference between healthy volunteers and patients, the heatmap in the middle corresponds to the median for each cytokine for each of the two populations studied and the column on the right is the *p*-value of statistical analysis using one-way ANOVA. Bottom: Box plot showing the concentration in pg/mL of each of the cytokines whose Z-Score is significantly different between healthy volunteers and patients. Statistical analysis was performed using an unpaired Mann–Whitney Wilcoxon test. n.s, not significant; *p < 0.05, **p < 0.01, ***p < 0.001, ****p < 0.0001

We first determined if GBM presence could impact peripheral immune parameters by comparing immune cells in the blood of healthy volunteers *versus* GBM patients using flow cytometry analyses. When looking at frequency of lymphoid cell subsets, we observed a decrease in all lymphoid cell subsets, i.e. CD4 and CD8 T cells and NKT cells, but not NK cells in GBM patients, in comparison with controls (Fig. 1A). When looking at T cell subsets, significant reduction was only observed in the naïve CD4 and naïve CD8 subsets (Fig. 1B, C). We observed an increase in the frequency of CD4 and CD8 Foxp3 regulatory T (Treg) cells in GBM patients (Fig. 1D). When looking at T helper and CD8 subsets (using intracellular cytokine labelling) we observed a significant increase in the Th1 and Tc1 subsets in GBM patients (Fig. 1E, F). When we looked at T-cell cytokine production using IFNγ, IL-2, TNFα and granzyme B labelling, we observed a decrease in cells that do not produce cytokines and an increase in poly-functional cells producing 3 or 4 cytokines in GBM patients (Fig. 1G and Supplementary Fig. 1A). When looking at CD8 T cells, we similarly observed a decrease in the number of non-functional or mono-functional CD8 T cells, and an increase in tri-or quadri-functional CD8 T cells (Fig. 1G and Supplementary Fig. 1B). When looking at checkpoint receptor expression on T cells, we observed more expression of PD-1 and TIGIT in CD4 and CD8 T cells in GBM patients, but no increase in Tim3 expression in comparison to controls (Fig. 1H, I).

For myeloid subsets, we observed an increase in neutrophils and a decrease in activated monocytes and granulocytic MDSC (gMDSC) in GBM patients (Fig. 1J). When looking at checkpoint ligands, we observed a decrease in PD-L1, CD111, CD155 and Galectin-9 in CD14^+^ myeloid cells from GBM patients (Fig. 1K).

Using bioplex assays testing 30 different cytokines in patient plasma (Supplementary Table 1), we observed that 10 cytokines were significantly more present in the plasma of GBM patients than in controls, including inflammatory signals like CXCL10, GM-CSF, IL-6, Eotaxin, and IL-1rα (Fig. 1L and Supplementary Fig. 1C).

Together, these data underline that GBM impacts on peripheral immune response with the presence of serum inflammatory response, a reduction in the number of CD4 and CD8 lymphoid cells but an increase in Treg and inflammatory Th1 and Tc1 cells, with high checkpoint expression. Myeloid cells were also affected, with an increase in neutrophils, and decreased expression of checkpoint inhibitor ligands.

### Influence of steroid use on peripheral blood immune phenotyping and serum cytokines in GBM patients

**Fig. 2:**
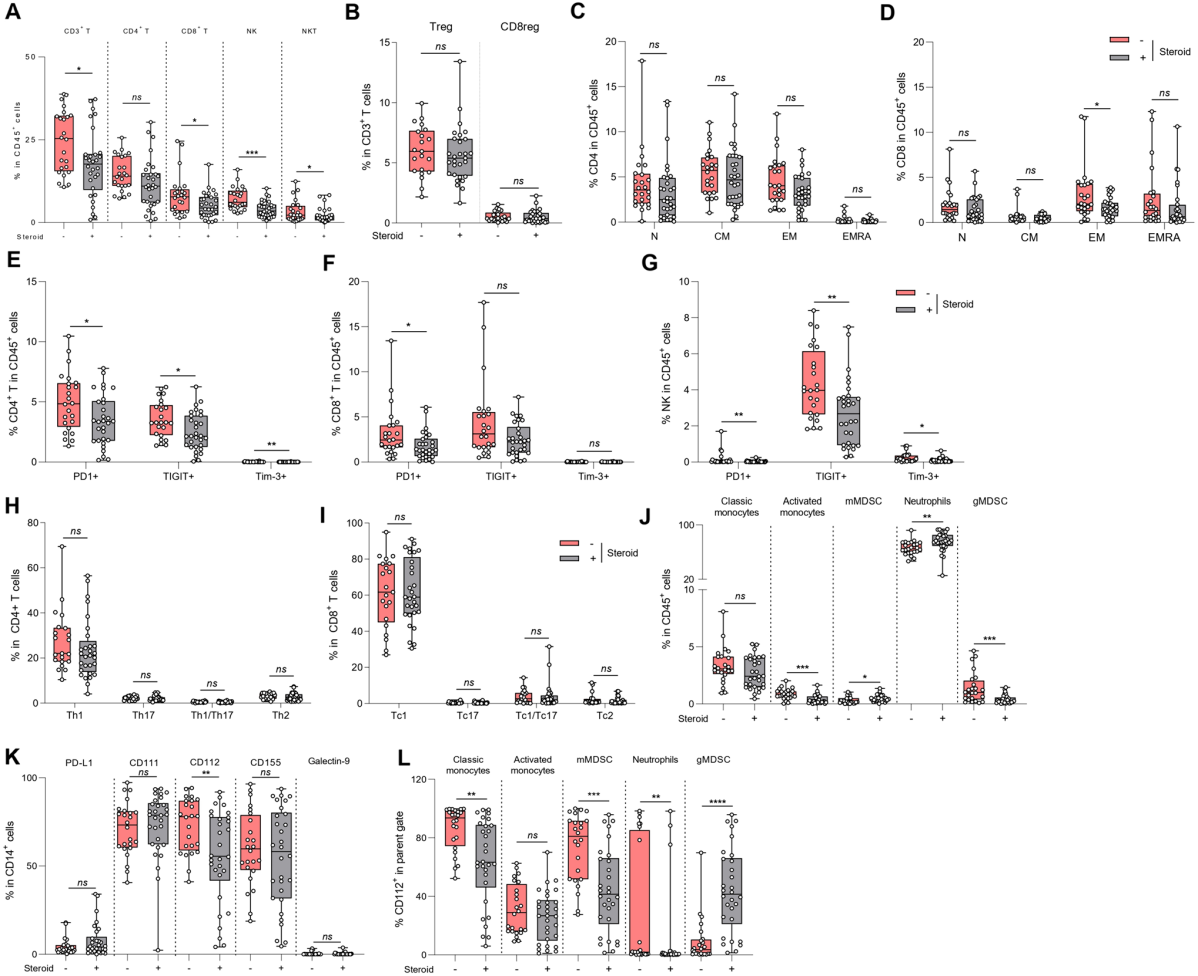
Influence of steroid usage peripheral blood immune phenotyping and serum cytokines in GBM patients. Blood samples from baseline glioblastoma patients were activated or not, stained and then analyzed by flow cytometry. Patients were distinguished according to the presence (n = 30) or the absence (n = 20) of steroid treatment at baseline (a-l). **A** Box plot showing the percentage of different lymphoid populations in CD45^+^ cells: CD4^+^ T cells (CD45^+^ CD3^+^ CD4^+^), CD8^+^ T cells (CD45^+^ CD3^+^ CD8^+^), Natural Killer (NK) cells (CD45^+^ CD3^−^ CD56^+^) and Natural Killer T (NKT) cells (CD45^+^ CD3^+^ CD56^+^) according to patients’ steroid treatment. **B** Box plot showing the frequency of regulatory CD4 T cells (CD4^+^ Foxp3^+^) and regulatory CD8 T cells (CD8^+^ Foxp3^+^) in lymphocytes T CD3^+^ according to patients’ steroid treatment. **C** Box plot showing the proportion of different stages of CD4 T cell differentiation in CD45^+^ cells: naïve cells « N» (CD4^+^ CD45RA^+^ CCR7^+^), effector memory cells « EM» (CD4^+^ CD45RA^−^ CCR7^−^), central memory cells « CM» (CD4^+^ CD45RA^−^ CCR7^+^) and effector memory cells re-expressing CD45RA « EMRA» (CD4^+^ CD45RA^+^ CCR7^−^) according to patients’ steroid treatment. **D** Box plot showing the proportion of different stages of CD8 T cell differentiation in CD45^+^ cells: naïve cells « N» (CD4^+^ CD45RA^+^ CCR7^+^), effector memory cells « EM» (CD4^+^ CD45RA^−^ CCR7^−^), central memory cells « CM» (CD4^+^ CD45RA^−^ CCR7^+^) and effector memory cells re-expressing CD45RA « EMRA» (CD4^+^ CD45RA^+^ CCR7^−^) according to patients’ steroid treatment. **E–G** Box plot showing the percentage of CD4^+^ T (**E**), CD8^+^ T (**F**) or NK (**G**) cells expressing 3 immune checkpoints PD-1, TIGIT or Tim-3 according to patients’ steroid treatment. **H** Box plot showing the proportion of different CD4 T cell subpopulations in CD4^+^ cells: Th1 (CD4^+^ FoxP3^−^ IFNγ^+^ IL-17A^−^), Th17 (CD4^+^ FoxP3^−^ IFNγ^−^ IL-17A^+^), Th1/17 (CD4^+^ FoxP3^−^ IFNγ^+^ IL-17A^+^) and Th2 (CD4^+^ FoxP3^−^ IFNγ^−^ IL-17A^−^ IL-4^+^) cells according to patients’ steroid treatment. **I** Box plot showing the proportion of different CD8 T cell subpopulations in CD8^+^ cells: Tc1 (CD8^+^ FoxP3^−^ IFNγ^+^ IL-17A^−^), Tc17 (CD8^+^ FoxP3^−^ IFNγ^−^ IL-17A^+^), Tc1/17 (CD8^+^ FoxP3^−^IFNγ^+^ IL-17A^+^) and Tc2 (CD8^+^ FoxP3^−^ IFNγ^−^ IL-17A^−^ IL-4^+^) cells according to patients’ steroid treatment. **J** Box plot showing the frequency of myeloid subpopulations in CD45^+^ cells: classical monocytes (CD11b^+^ CD15^−^ CD14^+^ HLA-DR^+^), activated monocytes (CD11b^+^ CD15^−^ CD14low HLA-DR^+^), monocytic MDSC (CD11b^+^ CD15^−^ CD14^+^ HLA-DR^low^), neutrophils (CD11b^+^ CD15^+^ CD14^−^) and granulocytic MDSC (CD11b^+^ CD15^+^ CD14^+^) according to patients’ steroid treatment. **K** Box plot showing the frequency of PD-L1^+^, CD111^+^, CD112^+^, CD155^+^ and Galectin-9^+^ populations within monocytes (CD14^+^) according to patients’ steroid treatment. **L** Box plot showing the frequency of CD112^+^ among the following myeloid subtypes: classical monocytes, activated monocytes, mMDSC, neutrophils and gMDSC according to patients’ steroid treatment. Statistical analysis was performed using an unpaired Mann–Whitney Wilcoxon test. n.s, not significant; *p < 0.05, **p < 0.01, ***p < 0.001, ****p < 0.0001

We next compared GBM patients treated with versus without steroids, and we observed that steroids induced a decrease in the frequency of total CD3 T cells and also in CD8 T cells (Fig. 2A). Steroid use also reduced the frequency of NK cells and NKT cells (Fig. 2A). When looking at Foxp3 expression on CD8 and CD4 regulatory T cells subsets, we observed that the frequency of Foxp3^+^ CD4 and CD8 Treg in GBM was independent of steroid use (Fig. 2B). Similarly, steroids did not affect the frequency of effector/memory subpopulations in CD4 T cells (Fig. 2C). However, steroid-treated GBM patients had significantly fewer effector memory CD8 T cells (Fig. 2D). Steroids induced a significant reduction in PD-1 on CD4, CD8 T cells and also on NK cells and a significant reduction in TIGIT on CD4 T and NK cells (Fig. 2E–G). However, steroid use did not impact T helper frequency or affect the functionality of Tc1 or Th1 cells (Fig. 2H, I).

Concerning the myeloid counterpart, significantly more myeloid cells were observed in patients treated with steroids. Patients treated with steroid presented a higher frequency of monocytic MDSC (mMDSC) and neutrophils, but had fewer granulocytic MDSC and activated monocytes (Fig. 2J). For checkpoint inhibitor receptors, we observed a significant reduction only in CD112 expression on myeloid cells in steroid-treated patients (Fig. 2K). When we looked at the expression of each of these checkpoint inhibitor receptors on the different myeloid subtypes, we observed that CD112 expression was reduced on classical monocytes, mMDSC and neutrophils, but increased on gMDSC in the presence of steroids (Fig. 2L). No differences were found in the other myeloid subtypes (Supplementary Fig. 2A–D).

With regard to serum cytokine levels, there was no difference between steroid-treated and non-steroid-treated patients (Supplementary Fig. 2E).

These data suggest that steroid use reduced the overall number of T cells and reduced checkpoint inhibitor expression, without affecting the functionality of remaining cells.

### Influence of MGMT status on peripheral blood immune phenotyping

**Fig. 3:**
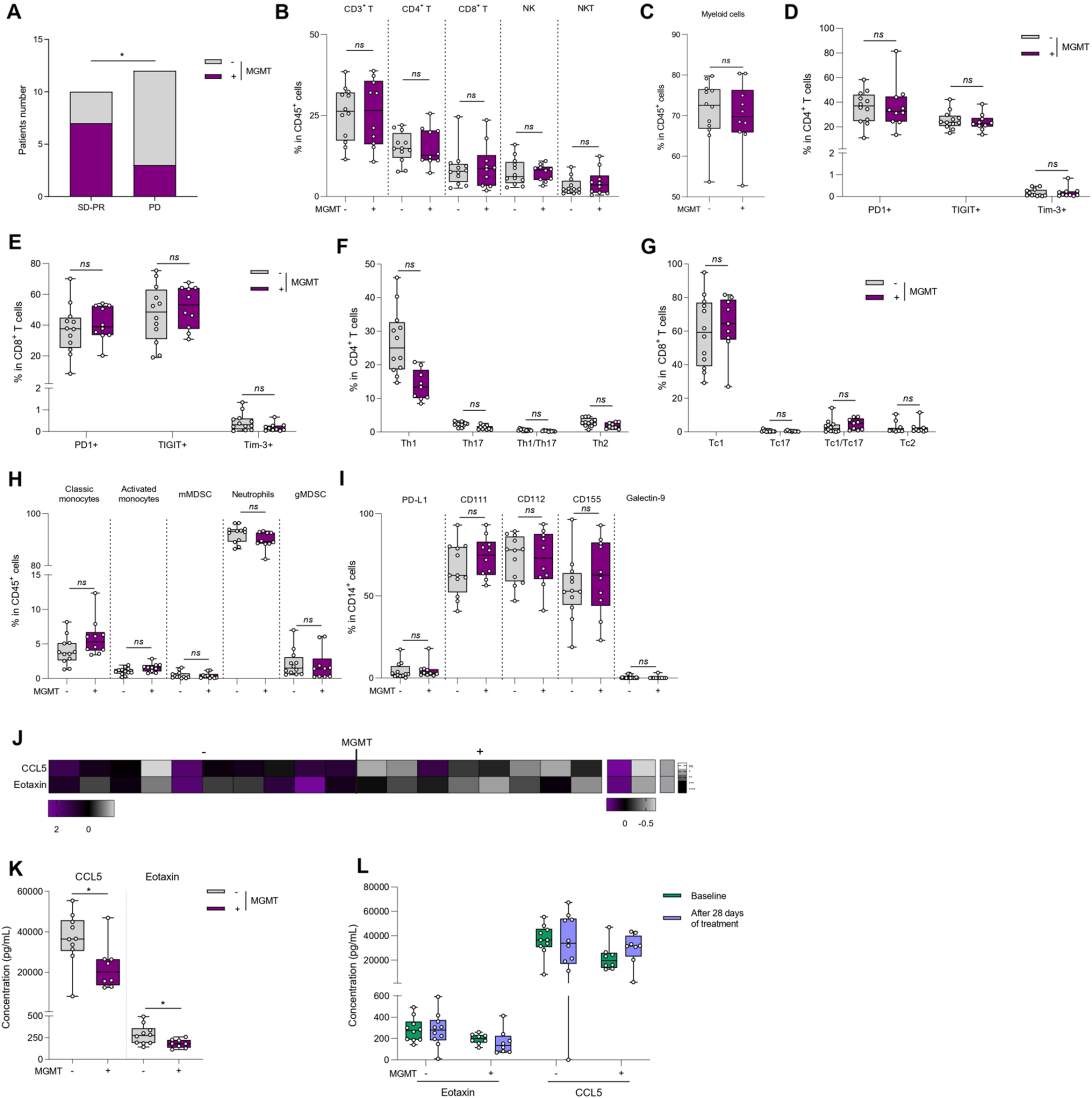
Influence of MGMT status on peripheral blood immune phenotyping. Blood samples from baseline glioblastoma patients were activated or not, stained and then analyzed by flow cytometry. Patients were distinguished according to the methylation (n = 10) or the absence of methylation (n = 12) of the MGMT promoter. **A** Bar plots showing the number of patients with stable disease or partial response (SD + PR) or progressive disease (PD) according to the methylation status of the MGMT promoter (+ : methylation of MGMT; −: absence of methylation of MGMT). *p < 0.05, comparison using Chi-square test. **B** Box plot showing the frequency of different lymphoid populations in CD45^+^ cells: CD4^+^ T cells (CD45^+^ CD3^+^ CD4^+^), CD8^+^ T cells (CD45^+^ CD3^+^ CD8^+^), Natural Killer (NK) cells (CD45^+^ CD3^−^ CD56^+^) and Natural Killer T (NKT) cells (CD45^+^ CD3^+^ CD56^+^) according to MGMT promoter status. **C** Box plot showing the percentage of myeloid cells (CD11b^+^) in CD45^+^ cells according to the status of MGMT promoter. **D, E** Box plot showing the percentage of CD4^+^ T (D) or CD8^+^ T (**E**) cells expressing 3 immune checkpoints PD-1, TIGIT or Tim-3 according to MGMT methylation status. **F** Box plot showing the proportion of different CD4 T cell subpopulations in CD4^+^ cells: Th1 (CD4^+^ FoxP3^−^ IFNγ^+^ IL-17A^−^), Th17 (CD4^+^ FoxP3^−^ IFNγ^−^ IL-17A^+^), Th1/17 (CD4^+^ FoxP3^−^ IFNγ^+^ IL-17A^+^) and Th2 (CD4^+^ FoxP3^−^ IFNγ^−^ IL-17A^−^ IL-4^+^) cells according to MGMT methylation status. **G** Box plot showing the proportion of different CD8 T cell subpopulations in CD8^+^ cells: Tc1 (CD8^+^ FoxP3^−^ IFNγ^+^ IL-17A^−^), Tc17 (CD8^+^ FoxP3^−^ IFNγ^−^ IL-17A^+^), Tc1/17 (CD8^+^ FoxP3^−^IFNγ^+^ IL-17A^+^) and Tc2 (CD8^+^ FoxP3^−^ IFNγ^−^ IL-17A^−^ IL-4^+^) cells according to MGMT methylation status. **H** Box plot showing the frequency of myeloid subpopulations in CD45^+^ cells: classical monocytes (CD11b^+^ CD15^−^ CD14^+^ HLA-DR^+^), activated monocytes (CD11b^+^ CD15^−^ CD14low HLA-DR^+^), monocytic MDSC (CD11b^+^ CD15^−^ CD14^+^ HLA-DR^low^), neutrophils (CD11b^+^ CD15^+^ CD14^−^) and granulocytic MDSC (CD11b^+^ CD15^+^ CD14^+^) according to MGMT methylation status. **I** Box plot showing the frequency of PD-L1^+^, CD111^+^, CD112^+^, CD155^+^ and Galectin-9^+^ populations within monocytes (CD14^+^) according to patients’ steroid treatment. **J** Cytokine secretion in patient plasma according to MGMT methylation status was analyzed using a bioplex technique. The heatmap on the left corresponds to normalized cytokine expression (z-score) for which there is a statistically significant difference between patients, the heatmap in the middle corresponds to the median for each cytokine for each of the two populations studied and the column on the right is the *p*-value of statistical analysis using one-way ANOVA. **K** Box plot showing the concentration in pg/mL of each of the cytokines whose Z-Score is significantly different between patients according to MGMT methylation status. **L** Box plots of the soluble Eotaxin and CCL5 assay in the plasma of patients at C1 (baseline), and C3 (after 28 days of treatment) according to MGMT methylation status. Statistical analysis was performed using an unpaired Mann–Whitney Wilcoxon test. n.s, not significant; *p < 0.05

Methylation of MGMT is a strong prognostic marker in GBM and is associated with intrinsic resistance to temozolomide [36]. However, the influence of MGMT status on baseline immune response in GBM remains unexplored. To avoid any bias, we focused this analysis only on patients not treated with steroids. As expected, MGMT methylated patients presented better response rates than MGMT demethylated patients (Fig. 3A). The frequency of lymphoid or myeloid cells were not affected by MGMT status (Fig. 3B, C). When looking at T cell subsets and checkpoint inhibitor expression, no differences were observed according to MGMT methylated status (Fig. 3D, E and Supplementary Fig. 3A, B). We did not observe any difference in T helper differentiation and Th1 cytokine secretion (Fig. 3F, G). Similarly, for myeloid cells, no differences were observed in the frequency of the different subsets or in the expression of checkpoint inhibitor receptors in methylated versus unmethylated MGMT patients (Fig. 3H, I and Supplementary Fig. 3C–G).

Analysis of serum cytokine levels showed that 2 chemokines (CCL5 and Eotaxin) were less present in the serum of MGMT methylated patients (Fig. 3J, K and Supplementary Fig. 3H). During radio-chemotherapy, the level of CCL5 and Eotaxin remained higher in unmethylated MGMT patients (Fig. 3L).

Together, these data show similar peripheral immune profiles in MGMT methylated and unmethylated patients.

### Evolution of immune parameters during therapy

**Fig. 4:**
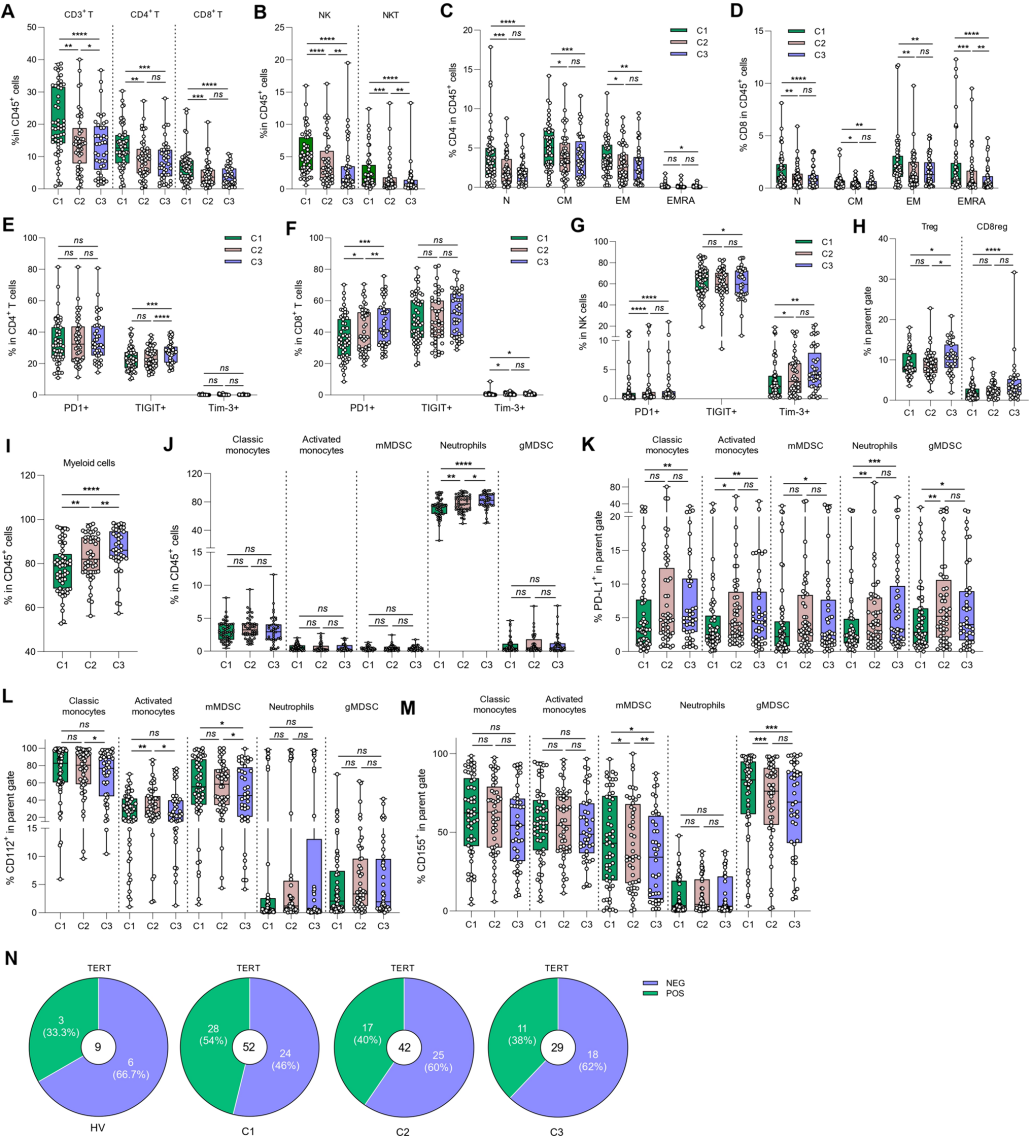
Evolution of immune parameter during therapy. Blood samples from glioblastoma patients were recovered at baseline (C1) (n = 54), 21 days (C2) (n = 46) and 35 days (C3) (n = 41) after the start of treatment and were activated or not, stained and then analyzed by flow cytometry. **A** Box plot showing the evolution of the percentage of different lymphoid populations in CD45^+^ cells: CD4^+^ T cells (CD45^+^ CD3^+^ CD4^+^) and CD8^+^ T cells (CD45^+^ CD3^+^ CD8^+^) during treatment. **B** Box plot showing the evolution of the percentage of different lymphoid populations in CD45^+^ cells: Natural Killer (NK) cells (CD45^+^ CD3^−^ CD56^+^) and Natural Killer T (NKT) cells (CD45^+^ CD3^+^ CD56^+^) during treatment. **C, D** Box plot showing the evolution of the proportion of different stages of CD4 (**C**) or CD8 (**D**) T cell differentiation in CD45^+^ cells: naïve cells «N» (CD4^+^ CD45RA^+^ CCR7^+^), effector memory cells «EM» (CD4^+^ CD45RA^−^ CCR7^−^), central memory cells « CM» (CD4^+^ CD45RA^−^ CCR7^+^) and effector memory cells re-expressing CD45RA «EMRA» (CD4^+^ CD45RA^+^ CCR7^−^). **E–G** Box plot showing the evolution of the percentage of CD4^+^ T (**E**), CD8^+^ T (**F**) or NK (**G**) cells expressing 3 immune checkpoints PD-1, TIGIT or Tim-3 during treatment. **H** Box plot showing the evolution of the frequency of regulatory CD4 T cells (CD4^+^ Foxp3^+^) and regulatory CD8 T cells (CD8^+^ Foxp3^+ ^) in lymphocytes T CD3^+^. **I** Box plot showing the evolution of the percentage of myeloid cells (CD11b^+^) in CD45^+^ cells during treatment. **J** Box plot showing the evolution of the frequency of myeloid subpopulations in CD45^+^ cells: classical monocytes (CD11b^+^ CD15^−^ CD14^+^ HLA-DR^+^), activated monocytes (CD11b^+^ CD15^−^ CD14low HLA-DR^+^), monocytic MDSC (CD11b^+^ CD15^−^ CD14^+^ HLA-DR^low^), neutrophils (CD11b^+^ CD15^+^ CD14^−^) and granulocytic MDSC (CD11b^+^ CD15^+^ CD14^+^) during treatment. **K–M** Box plot showing the frequency of PD-L1^+^ (**K**), CD112^+^ (**L**) or CD155^+^ (**M**) among the following myeloid subtypes: classical monocytes, activated monocytes, mMDSC, neutrophils and gMDSC during treatment. **N** Parts of whole (purple and green) showing the percentage of positive (in green) or negative (in purple) antitumor responses against TERT in healthy volunteers (n = 9) and in patients at C1 (n = 52), C2 (n = 42) and C3 (n = 29). Statistical analysis was performed using an unpaired Mann–Whitney Wilcoxon test. n.s, not significant; *p < 0.05, **p < 0.01, ***p < 0.001, ****p < 0.0001

We then attempted to decipher the role of radio-chemotherapy on the evolution of blood immune parameters. To do this, we tested the frequency and function of both lymphoid and myeloid cells at baseline (C1), 14 (C2) and 28 (C3) days after the start of radio-chemotherapy. We observed a decrease in the frequency of all lymphoid cell subsets (CD3, CD4, CD8, NK, NKT cells) (Fig. 4A, B). Naïve, central memory, effector memory and effector memory expressing CD45RA^+^ CD4 T cells also significantly decreased during treatment (Fig. 4C). The same phenomenon was observed for CD8 T subtypes, with the same significant decrease for all T subsets (Fig. 4D). We observed an increase in TIGIT expression on CD4 T cells during treatment, and an increase in the expression of PD-1 and Tim3 in both CD8 and NK cells (Fig. 4E–G). The proportion of CD4 Tregs was significantly increased at timepoint C3, whereas the proportion of CD8 regulatory cells was enhanced since the beginning of treatment (Fig. 4H). Concerning the functionality of T cells, treatment induced no modification of CD4 T helper and CD8 T cytotoxic cell subsets, apart from a minor increase in the Th17 cell population (Supplementary Fig. 4A, B). At the level of myeloid cells, treatment increased the proportion of myeloid cells, mainly driven by a higher frequency of neutrophils (Fig. 4I, J). The increase in the proportion of neutrophils was matched by an increase in the expression of PD-L1 on their surface (Fig. 4K). We also observed an increase in PD-L1 expression in all other myeloid subtypes (Fig. 4K). Conversely, we observed a decrease in CD112 expression on the surface of classic, activated monocytes and mMDSCs, and a decrease in CD155 on the surface of monocytic and granulocytic MDSCs (Fig. 4L, M). No change in the expression of CD111 or galectin 9 was observed on myeloid cells (Supplementary Fig. 4C, D). Analysis of serum cytokine levels showed that IL-15 concentration decreased during treatment, reflecting the disappearance of signals for T and NK cell expansion and maturation (Supplementary Fig. 4E).

To estimate the specific anti-tumor response of T cells against GBM, we tested the immune response against the shared tumor antigen Telomerase. Telomerase is highly expressed in most GBM because of TERT promoter mutation, which is observed in more than 80% of GBM. As previously described, we used the UCP peptides, which bind to most HLA class II molecules. We observed a greater TERT-specific CD4 response in GBM patients (54%) compared with healthy volunteers (33.3%) (Fig. 4N). However, we observed a decrease in the frequency of patients with a specific TERT response during treatment (40% at C2 and 38% at C5) (Fig. 4N).

These data reflect the fact that radio-chemotherapy induces a decrease in lymphoid cells and an increase in the proportion of T and NK cells with exhausted function, and a proportional increase in neutrophils. In addition, at serum level, IL-15, a factor known to induce expansion and maturation of T and NK cells, decreased during treatment, confirming the harmful effect of chemotherapy on the adaptive immune response.

### Relation between immune parameters and prognosis

**Fig. 5:**
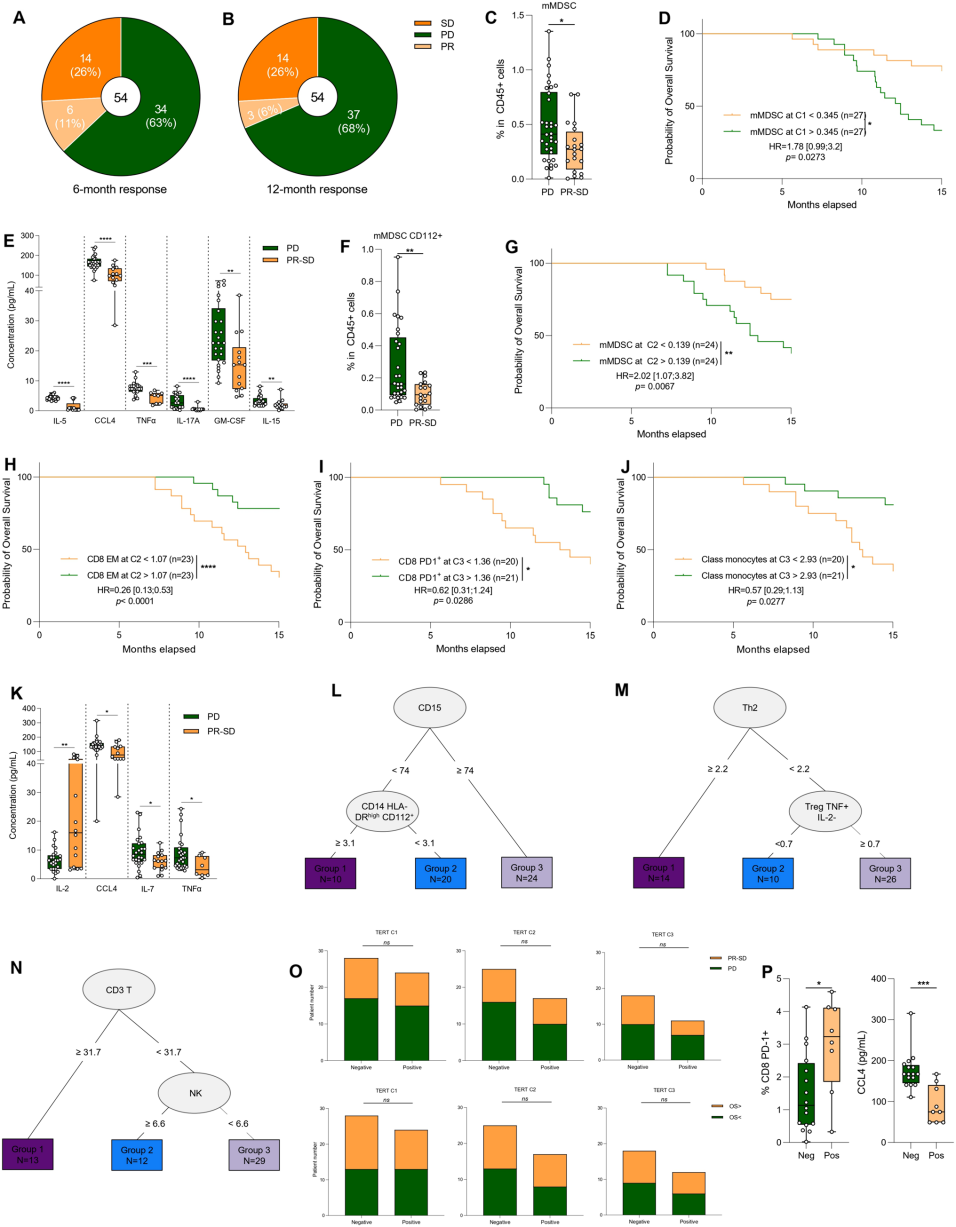
Relation between immune parameter and prognosis. **A, B** Parts of whole (light and dark orange and green) showing the percentage of patients’ response: progression disease (PD, in green), stable disease (SD, in dark orange) or partial response (PR, in light orange) at six months (**A**) or 12 months (**B**). **C** The frequency of mMDSC (CD11b^+^ CD15^−^ CD14^+^ HLA-DR^low^) at baseline according to response status (progression disease *versus* partial response + stable disease) is depicted (n = 54). **D** Kaplan–Meier curves for overall-free survival with patients stratified according to baseline mMDSC frequency (n = 54). The overall median was used as a threshold to distinguish the two groups. Two-sided P value with significance level set at 0.05. **E** Box plot showing the concentration at baseline in pg/mL of IL-5, CCL4, TNFα, IL-17A, GM-CSF, IL-15 according to response status (progression disease *versus* partial response + stable disease). A z-score analysis revealed a significant difference between responder (SD + PR) and non-responder (PD) patients. **F** The frequency of mMDSC CD112^+^ at C2 according to response status (progression disease *versus* partial response + stable disease) is depicted (n = 48). **G** Kaplan–Meier curves for overall-free survival with patients stratified according to mMDSC frequency at C2 (n = 48). The overall median was used as a threshold to distinguish the two groups. Two-sided P value with significance level set at 0.05. **H–J** Kaplan–Meier curves for overall survival with patients stratified according to CD8 PD-1^+^ (**H**), CD8 Effector Memory (**I**) or classical monocytes (**J**) frequency at C3 (n = 41). The overall median was used as a threshold to distinguish the two groups. Two-sided P value with significance level set at 0.05. **K** Box plot showing the concentration at C3 in pg/mL of IL-2, CCL4, IL-7, TNFα according to response status (progression disease versus partial response + stable disease). A z-score analysis revealed a significant difference between responder (SD + PR) and non-responder (PD) patients. **L–N** Decision tree for progression-free survival estimated with myeloid panel (**L**), cytokine panel (**M**) or lymphoid panel (**N**) parameters. **O** Top: Bar plots showing the number of partial response + stable disease (PR + SD) or progression disease (PD) patients according to TERT-specific T-cell responses at C1 (left), at C2 (middle) and at C5 (right). Bottom: Bar plots showing the patients number stratified with overall survival according to TERT-specific T-cell responses at C1 (left), at C2 (middle) and at C5 (right).Two-sided P value with significance level set at 0.05, comparison using Fisher’s exact test. **P** PD-1^+^ CD8 percentage (left) and plasma CCL4 levels according to TERT antitumor T-cell response negative (Neg) or positive (Pos) in glioblastoma patients at baseline. Statistical analysis was performed using an unpaired Mann–Whitney Wilcoxon test. n.s, not significant; *p < 0.05, **p < 0.01, ***p < 0.001, ****p < 0.0001. Survival distributions were compared using the log-rank test (**D**, **G**–**J**)

In our cohort, 34 patients experienced disease progression within six months of treatment, while 20 patients were considered good responders (partial response or stable disease) with more than six months of disease control (Fig. 5A). At 12 months, 37 patients had progressive disease whereas 17 patients had a good response (Fig. 5B). We looked for immune markers associated with disease progression in the year following the start of treatment. To do this, 162 clinical and biological parameters were tested. To avoid bias due to multiple statistical tests, we performed both univariate and multivariate logistic analyses to find biomarkers associated with response to treatment.

At baseline, only a high level of mMDSC was associated with disease progression and poor outcome (Fig. 5C, D). We also observed higher levels of IL-5, CCL4, TNFα, IL-17A, GM-CSF, IL-15 cytokines at baseline in the serum of patients with progressive disease (Fig. 5E and Supplementary Fig. 5A).

At C2, mMDSC expressing CD112 were also associated with disease progression and poor outcome (Fig. 5F, G). Conversely, a high proportion of CD8, particularly memory CD8 effectors, and a high level of NK cells were associated with better outcome (Fig. 5H and Supplementary Fig. 5B, C). At C3, a high level of CD8 expressing PD-1 and a high proportion of classic monocytes were associated with better outcome (Fig. 5I, J). At serum level, at C3, we found that higher levels of CCL4 and TNFα were present in patients with progressive disease. Conversely, responders had higher serum levels of IL-2 (Fig. 5K and Supplementary Fig. 5D).

To further explore the prognostic role of the lymphoid and myeloid compartments and T-cell functionality, we used a decision tree to generate biomarkers of response at baseline. We generated 3 independent biomarkers based on each individual flow cytometry analysis. All variables were studied accordingly to progression-free survival (PFS) to determine potential prognostic marker associations. CD15 level, CD112^+^ classic monocytes, Th2 cells, Treg cells expressing TNFα, CD3 T cells and NK cells proportion were retained as the most important variables to predict outcome (Fig. 5L–N and Supplementary Fig. 5E–G). Each cytometry test using a lymphoid, myeloid and functionality panel could select of population of patients with prolonged PFS. When looking at CD4 tumor specific immune response, we observed no difference in TERT-specific T response at baseline between responders and non-responders (Fig. 5O). During treatment, there was also no difference in TERT-specific T response between responder and non-responder patients (Fig. 5O). However, we found that patients with positive TERT response exhibited a high level of CD8 T cells expressing PD-1 and a low level of CCL4 (Fig. 5P), markers related to progression-free survival.

Together these data suggest that baseline immune blood parameters are efficient to predict PFS and prolonged response to radio-chemotherapy in patients with GBM.

## Discussion

Our study provides new insights into the impact of GBM on peripheral immune parameters, the influence of steroid use, MGMT status and radio-chemotherapy on immune response, and the relationship between immune parameters and prognosis in GBM patients treated with radio-chemotherapy.

Our study demonstrates that GBM is associated with a significant alteration of peripheral immune parameters. We observed a decrease in the total frequency of lymphoid cells and an increase in neutrophils in GBM patients compared with controls. This is consistent with previous studies showing that GBM can induce systemic immune suppression. At the level of the lymphoid compartment, the sequestration of T cells in the bone marrow of patients with brain tumors was previously reported, and could explain the lower number of CD8 and CD4 T cells [20]. In contrast, we report an increase in neutrophils and a decrease in activated monocytes in GBM patients. Previous reports showed downregulation of HLA-DR on circulating monocytes and an increased number of circulating CD33^+^ HLA-DR^−^ myeloid-derived suppressor cells (MDSCs) comprised of immature, monocytic and neutrophilic subsets [9, 37] thus corroborating our data. We also found that GBM patients have an increased number of Treg cells. Similar Treg accumulation were also previously described and was associated with poor prognosis [38]. The mechanism remains to be elucidated, but previous data suggest that PD-L1-expressing myeloid cells or GARP expression could be involved in Treg expansion [39, 40]. The expression of immune checkpoints and T cell functionality, on the other hand, have been little analyzed in the context of GBM. Our data support the accumulation of T cells with an exhausted phenotype, and conserved Th1/Tc1 function, thus suggesting an association between GBM and induction of the peripheral immune response. The higher presence of telomerase-specific T cells in GBM patients compared to controls patients is also an argument for the presence of a spontaneous immune response against GBM [34]. However, analysis of telomerase-specific immune response did not find any association between immune response and outcome. These findings suggest that GBM may promote an immunosuppressive response that favors tumor growth and progression, and may limit the efficacy of the antitumor immune response.

Steroids are commonly used to treat GBM. We found that steroid use was associated with a further decrease in the number of CD3 T cells, CD8 T cells, and NK cells, while increasing the number of neutrophils. Steroids also induced a decrease in the expression of PD-1 and TIGIT on CD4 and CD8 T cells. These findings suggest that steroids may further compromise the immune response in GBM patients. Retrospective clinical analyses have indeed identified corticosteroid use during first-line radiotherapy as an independent indicator of shorter survival in three independent cohorts of GBM patients [41]. Patients receiving steroids had higher neutrophil counts. This is a known effect of steroid use, due to neutrophil demargination from the endovascular lining of the blood vessels [42]. The decrease in T cells mainly involves naïve populations. In mice, it has been shown that dexamethasone, a synthetic steroid, induced apoptosis of naïve and memory CD8^+^ T cells without affecting effector cells. While in this model, steroids compromised anti-viral immunity through reductions in the naïve cell pool, it is plausible that steroids negatively affect anti-GBM immune response in the same way [43].

MGMT promoter methylation is a strong prognostic biomarker in GBM [36]. We found that MGMT methylation status did not have a significant impact on the frequency of lymphoid or myeloid cells in GBM patients. However, MGMT methylated patients had higher expression of CD111 and PD-L1 on myeloid cells. These findings suggest that MGMT methylation is not involved in the adaptive immune response; however, this study suggests that MGMT status probably affects the activation of myeloid cells in GBM patients.

We found that radio-chemotherapy was associated with a further decrease in the number of lymphoid cells, particularly CD4 T cells and CD8 T cells. Radio-chemotherapy also induced an increase in the expression of PD-1 and TIGIT on CD4 and CD8 T cells, and an increase in the expression of PD-L1 on myeloid cells, thus suggesting that radio-chemotherapy impedes the adaptive immune response while preserving exhausted T cells. Temozolomide is a well-known immunosuppressive chemotherapy [44] with the ability to deplete the B and T cell compartments. Our data corroborate the deleterious effect of temozolomide on T cells and telomerase immune response. The effect of temozolomide on T cell exhaustion is poorly known. In mice models of GBM, upregulation of markers of T cell exhaustion such as LAG-3 and Tim3 in lymphocytes has been observed with temozolomide, and was associated with the resistance to immunotherapy, thus probably inducing terminal differentiation of exhausted T cells [45]. Our data corroborate these preclinical data and suggest that radio-chemotherapy may suppress the immune response in GBM patients by eliminating functional T cells, leading to an accumulation of exhausted T cells in terminal differentiation.

Our study revealed a strong association between immune parameters and prognosis. Despite the inefficacy of current immunotherapies in GBM, this is a strong argument in favour of the hypothesis that the immune context might modulate patient outcome. Notably, we observed that a high level of mMDSC, Th2 cells and Treg cells, and a low level of CD3 T cells and NK cells were associated with poor prognosis. Because of the well-known immune role of these populations in cancer immune response, these data are logical. Previous studies support the finding that Tregs, neutrophils and MDSC are associated with outcome in GBM, and more precisely that a high level of one of these populations is associated with reduced survival [46, 47]. Similarly, in a multi-cancer cohort; blood immune profiles; involving the analysis of lymphoid and myeloid cells, were found to be associated with outcome [48]. Our study reinforces previous observations from a homogenous cohort of patients all treated with standard of care radio-chemotherapy [48]. Taken together, all these findings suggest that the immune response may play an important role in determining the outcome of GBM patients treated with radio-chemotherapy.

Our study has limitations due to the single-centre design, the relatively small sample size and the high proportion of males, all of which limit the generalizability of the conclusions. Future studies with larger, more diverse populations are needed to confirm our findings. Additionally, our study did not assess intra-tumoral immune response and the concordance between blood and intra-tumoral parameters remains to be explored.

## Conclusion

Our study provides new insights into the complex relationship between GBM and the immune system. GBM is associated with a significant alteration of peripheral immune parameters at baseline, with stigmata of immunosuppression despite the presence of an inflammatory anticancer immune response. Moreover, these alterations are further exacerbated by steroid use and radio-chemotherapy. We also show that several of these baseline immune parameters are associated with poor prognosis in these patients. These findings suggest that targeting the immune system and especially immunosuppression may be a promising strategy for improving the treatment of GBM.

### Supplementary Information

Below is the link to the electronic supplementary material.

Supplementary file1 (PDF 453 KB)

Supplementary file2 (PDF 439 KB)

## Data Availability

Data are available upon reasonable request.
