# Supplementary material for: Effect of radiochemotherapy on peripheral immune response in glioblastoma
Source: Cancer Immunol Immunother. 2024 May 16;73(7):133. doi: 10.1007/s00262-024-03722-5 (PMC11098987; doi:10.1007/s00262-024-03722-5)
Supplement: Supplementary file 1 — Supplementary file1 (PDF 453 KB) [file 262_2024_3722_MOESM1_ESM.pdf]

Supplementary material

Supplementary methods

Serum isolation

In order to isolate the serum, a serum separator tube of blood was centrifuged at 1000g for 10 min. The serum was then gently removed and stored at 500µL per cryotube at -80°C.

Peripheral blood mononuclear cell isolation

PBMC were isolated from whole blood by density gradient centrifugation (Lymphocyte Separation Medium, CMSMSL0101, Eurobio) with SepMate tubes (85460, Stemcell Technologies). Whole blood was transferred into Sepmate tubes at a rate of 17 mL of whole blood per tube and then centrifuged at 1200g for 10 min with an acceleration of 5 and the brake off. After removing as much plasma as possible, the phase containing the enriched PBMCs could be recovered. After washing with 45 mL PBS, centrifugation of 300g for 7 min was carried out and the PBMC pellet was resuspended in 5 mL PBS 1x for counting. Then, a final wash with 10 mL of PBS 1x was performed before cryopreservation, which consists in freezing at a rate of 8.10^6 cells per cryotube in a solution of 50% Foetal Bovine Serum (FBS, Dutscher), 40% RPMI 1640 (L0500-500, Dutscher) and 10% DMSO (P60-36720100, Dutscher) until further use.

Cytokine measurement

Forty-five analytes were quantified in the plasma using Human XL Cytokine Magnetic 45-plex Luminex® assay (#898855, R&D Systems, USA) according to the manufacturer's instructions. The performance assay standard values for each analyte are detailed in Extended Data Table 1. Briefly, the plasma aliquots were thawed and diluted by half with the diluent provided in the kit. Reagents and standards were also prepared before the technique was started. The samples

were then mixed with the antibody-bound magnetic beads in a 96-well plate and incubated overnight at 4°C with agitation. The following day, the plate was washed several times using a BioPlex Pro II Wash Station. The plate was then incubated for 1 hour at room temperature with agitation with the biotinylated detection antibody and then for 30 minutes with streptavidin-PE. After washing the plate, the buffer provided in the kit was added to the wells before reading the plate on the Bioplex 200 (Biorad). The results were then analyzed using the BioPlex Manager software.

Cytometry analysis

- Lymphoid and myeloid population identification

Antibodies for lymphoid cell analysis: Multicolour flow cytometry was performed using Beckman Coulter's custom design service and its dry coating technology, custom tubes containing anti-CD56-ECD (clone N901), anti-CD45-PECy5.5 (clone J.33), anti-PD-1-APC (clone PD1.3), anti-CD3-AA750 (Clone UCTH1), anti-CD45RA-PacBlue (clone 2H4LDH11LDB9), anti-CD8-KromeOrange (Clone B9.11) and a mortality marker DRAQ7 were produced. The following liquid antibodies were added to the custom tubes: anti-DNAM-1-FITC (BioLegend, clone TX25), anti-CD96-PE (BioLegend, clone NK92.39), anti-TIGIT-PECy7 (BioLegend, clone A15153G), anti-CCR7-BV605 (BioLegend, clone G043H7), anti-Tim-3-BV650 (BioLegend, clone F38-E2E2) and anti-CD4-BV785 (BioLegend, clone OKT4). Antibodies for myeloid cell analysis: Multicolour flow cytometry was also performed using Beckman Coulter's custom design service and its dry coating technology, custom tubes containing anti-CD11b-FITC (clone Bear1), anti-HLA-DR-ECD (clone Immu-357), anti-PD-L1-APC (clone PDL1.3.1), anti-CD15-PacBlue (clone 80H5), anti-CD14-KromeOrange (clone RMO52) and a mortality marker DRAQ7 were produced. The following liquid antibodies were added to the custom tubes: anti-CD111-PE (BioLegend, clone R1.302), anti-CD155-PerCPCy5.5 (BioLegend, clone SKIL4), anti-CD112-PC7 (BioLegend, clone TX31), anti-

CD163-APCCy7 (BioLegend, clone GHI/61), anti-CD3-BV605 (BioLegend, clone OKT3), anti-CD19-BV605 (BioLegend, clone HIB19), anti-CD20-BV605 (BioLegend, clone 2H7), anti-CD56-BV605 (BioLegend, clone HCD56), anti-Galectin-9-Biotin (Miltenyi Biotec, clone RG9-35.7), anti-Streptavidin-BV650 (BioLegend) and anti-CD45-BV785 (BioLegend, clone HI30).

- Lymphocyte function analysis

Using Beckman Coulter's custom design service and its dry coating technology, custom tubes containing anti-IFNγ-FITC (clone 45.15), anti-TNFα-PE (clone IPM2 (188)), anti-IL-4-PECy7 (clone MP4-25D2), anti-Foxp3-AF647 (Clone 259D), anti-IL-17A-AF700 (Clone BL168), anti-CD3-AA750 (clone UCHT1), anti-CD4-PacBlue (clone 13B8.2) and anti-CD8-KromeOrange (clone B9.11) were produced. Liquid antibodies were also used: anti-GranzymeB-PECy5.5 (ThermoFisher, clone GB11), anti-IL-2-BV650 (BioLegend, clone MQ1-17H12) and anti-CD45-BV785 (BioLegend, clone HI30).
