# Supplementary material for: Effect of radiochemotherapy on peripheral immune response in glioblastoma
Source: Cancer Immunol Immunother. 2024 May 16;73(7):133. doi: 10.1007/s00262-024-03722-5 (PMC11098987; doi:10.1007/s00262-024-03722-5)
Supplement: Supplementary file 2 — Supplementary file2 (PDF 439 KB) [file 262_2024_3722_MOESM2_ESM.pdf]

Supplementary File Table 1. List of cytokines analyzed by Luminex Assay

Cytokine	Assay Working range (pg/ml)
CD40 Ligand	1659 - 59,995
EGF	32.6 - 1102
Eotaxin	63.9 - 2299
FGF basic	34.0 - 1084
Flt-3 Ligand	81.7 - 2783
G-CSF	29.8 - 1008
GM-CSF	60.9 - 2069
Granzyme B	14.3 - 469
Groα	173 - 2264
Groβ	38.3 - 1247
IFNα	14.7 - 500
IFNβ	13.0 - 446
IFNγ	18.1 - 611
IL-1α	36.0 - 1199
IL-1β	12.2 - 432
IL-1ra	41.9 - 1386
IL-2	12.9 - 433
IL-3	61.0 - 2093
IL-4	4.10 - 140
IL-5	22.0 - 780
IL-6	34.3 - 1161
IL-7	14.1 - 484
IL-8	5.31 - 184
IL-10	102 - 2997
IL-12 p70	72.7 - 2419
IL-13	111 - 3847
IL-15	7.86 - 274
IL-17A	29.0 - 979
IL-17E	58.3 - 1938
IL-33	60.4 - 1926
IP-10	9.52 - 334
MCP-1	45.1 - 626
MIP-1α	42.9 - 571
MIP-1β	319 - 11,240
MIP-3α	9.08 - 307
MIP-3β	14.5 - 483
PDGF-AA	15.8 - 537
PDGF-AB/BB	24.6 - 867
PD-L1	271 - 9672
RANTES	1198 - 36,884
TGF-α	31.8 - 1098
TNFα	34.8 - 1325
TRAIL	87.3 - 2972
VEGF	19.9 - 678
